# Prevailing Negative Soil Biota Effect and No Evidence for Local Adaptation in a Widespread Eurasian Grass

**DOI:** 10.1371/journal.pone.0017580

**Published:** 2011-03-29

**Authors:** Viktoria Wagner, Pedro M. Antunes, Michael Ristow, Ute Lechner, Isabell Hensen

**Affiliations:** 1 Institute of Biology/Geobotany and Botanical Garden, Martin Luther University Halle-Wittenberg, Halle, Germany; 2 Department of Biology, Algoma University, Sault Ste. Marie, Ontario, Canada; 3 Institute of Biology/Plant Ecology and Nature Conservation, University of Potsdam, Potsdam, Germany; 4 Institute of Biology/Microbiology, Martin Luther University Halle-Wittenberg, Halle, Germany; Duke University, United States of America

## Abstract

**Background:**

Soil biota effects are increasingly accepted as an important driver of the abundance and distribution of plants. While biogeographical studies on alien invasive plant species have indicated coevolution with soil biota in their native distribution range, it is unknown whether adaptation to soil biota varies among populations within the native distribution range. The question of local adaptation between plants and their soil biota has important implications for conservation of biodiversity and may justify the use of seed material from local provenances in restoration campaigns.

**Methodology/Principal Findings:**

We studied soil biota effects in ten populations of the steppe grass *Stipa capillata* from two distinct regions, Europe and Asia. We tested for local adaptation at two different scales, both within (ca. 10–80 km) and between (ca. 3300 km) regions, using a reciprocal inoculation experiment in the greenhouse for nine months. Generally, negative soil biota effects were consistent. However, we did not find evidence for local adaptation: both within and between regions, growth of plants in their ‘home soil’ was not significantly larger relative to that in soil from other, more distant, populations.

**Conclusions/Significance:**

Our study suggests that negative soil biota effects can prevail in different parts of a plant species' range. Absence of local adaptation points to the possibility of similar rhizosphere biota composition across populations and regions, sufficient gene flow to prevent coevolution, selection in favor of plasticity, or functional redundancy among different soil biota. From the point of view of plant - soil biota interactions, our findings indicate that the current practice of using seeds exclusively from local provenances in ecosystem restoration campaigns may not be justified.

## Introduction

Soil biota effects are increasingly recognized as an important factor driving the abundance and distribution of plants [1]–[3]. These reciprocal interactions can result in different functional outcomes; from neutral, mutualistic to parasitic [4], [5]. By influencing seedling germination, plant growth and fitness, soil biota effects are an important factor controlling community composition, species diversity, and successional dynamics [6]–[9].

There is accumulating evidence that the functional outcomes of plant and soil biotic interactions within a habitat derive from coevolved relationships. This is supported by studies on invasive species, in which plant performance was compared in soil from within (‘home soil’) or outside the distribution range (‘away soil’). Several species were shown to be suppressed by their native ‘home soil’ and to be released from its negative effects when grown in soil from the introduced range, e.g. *Centaurea maculosa* [10], *Ammophila arenaria* [11], *Carpobrotus edulis* and *Carpobrotus*×cf. *acinaciformis* [12], and *Tragopogon dubius* [13]. Exotic plants can also be limited in their naturalization if suitable symbionts are not available in the introduced range, as documented for *Cytisus scoparius* [14], and Pinaceae species [15].

While these studies indicate that plants have coevolved with their native soil biota, it is less clear to what extent local adaptation varies among populations within the native range of a plant species [4], [16]–[19]. Evidence for local adaptation to soil biota was found in the case of *Trifolium repens* and *Rhizobium* bacteria [20], *Amphicarpaea bracteata* and *Bradyrhizobium* bacteria [21], *Allium vineale* and *Uniola paniculata* to arbuscular mycorrhizal fungi [22], [23]. Contrasting results have been reported for *Acacia* species and *Rhizobium* bacteria [24], *Pinus* and *Rhizopogon* fungi [25], *Ammophila arenaria* and nematodes [26]. Most of these studies were however limited because they did not consider the hypothesis that adaptation might be manifested at different spatial scales [27]–[29]. Generally, these studies were confined to specific organism groups whose effects may be overridden by more important soil organism groups under field conditions [13]. Furthermore, they tested for direct interactions between pairs of species, leaving out putative additive or net effects in interactions with soil biota.

The question of local adaptation between plants and their soil biota has far-reaching implications for studies on plant-soil biota interactions and land management. In restoration campaigns, for example, much effort is spent in the common practice of using seed material from local provenance [30]. If local adaptation between plants and soil biota exists, this would be one more justification in favor of this effort [31].

In this study, we tested whether plants are locally adapted to their soil biota by carrying out a reciprocal transplant experiment in the greenhouse; a recommended approach to examine local adaptation [32]. We used plant and soil material from populations located in two different regions of a species' range, and addressed our question at two spatial levels, within (ca. 10–80 km) and between (ca. 3300 km) two regions. We chose the steppe grass *Stipa capillata* L. as a model species because *S. capillata* is an important element of Eurasian dry grasslands [33] and grasses have been shown to be sensitive to soil biota [2]. Furthermore, this species has a wide distribution range in Eurasia, which allows testing for local adaptation at a large scale [34]. We used seed and soil material from populations in the centre of its distribution range, in Asia, and from the range periphery, in Europe. Peripheral populations were spatially and genetically more isolated than those in the central range (V. Wagner, W. Durka, I. Hensen, unpublished data), increasing the possibility for local coevolution with soil biota. Furthermore, as the two regions were 3300 km apart and differed climatically [34] we hypothesized local adaptation to be stronger between than within regions.

## Materials and Methods

### Study Species

**Figure 1:**
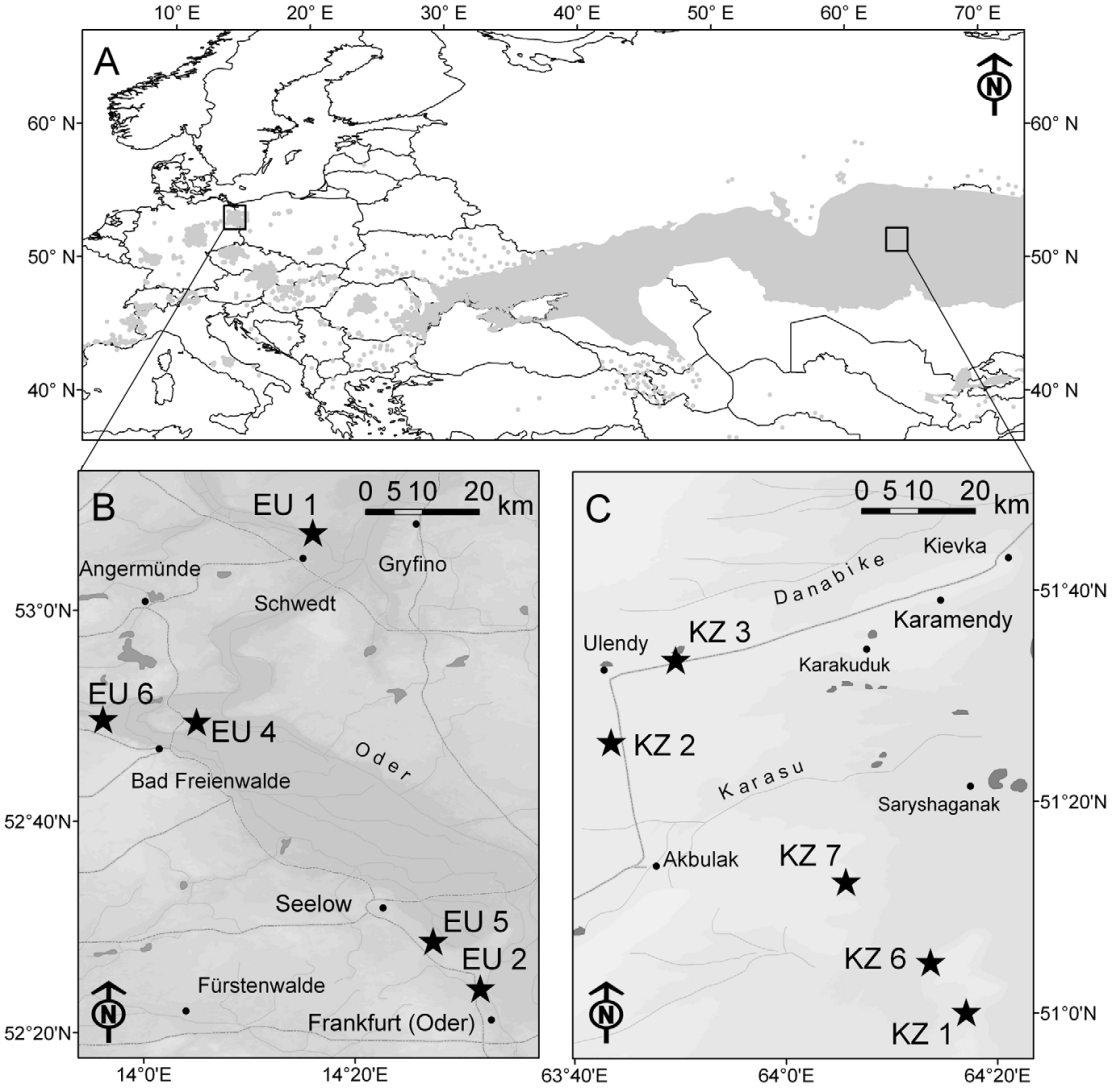
Map of the study localities. A) Overview of the study regions within the native distribution range of *Stipa capillata* (grey color). Detailed map of the study regions in (B) Europe and (C) Asia. Population localities are marked as black stars.

*Stipa capillata* L. (Poaceae) is a perennial tussock grass that grows in dry grasslands on nutrient poor, sandy to loamy soil. Its native range covers large areas of Eurasia and, in the core, in the steppes of Russia and Kazakhstan (Figure 1a), it is one of the most common plant species [33]. However, at its north-western range periphery in Central Europe (Figure 1a) *S. capillata* is rare, isolated and red-listed [34]. The species is recognized by its inflorescence, bearing single stalked florets with a long and naked awn. Flowers are known to be facultative cleistogamous [35]. Caryopses ripen and are dispersed in animal fur or by wind in late August to October. Mycorrhizal colonization in roots of *S. capillata* was reported from Russia by Mukhin and Betekhtina [36] and from China by Shi et al. [37].

### Study Sites

We collected seeds and soil from five populations within each of the two regions (i.e., Europe and Asia) that were approximately 3300 km apart. European populations, in the lower Oder basin, in Brandenburg, Germany (Figure 1B) were 9.7–82 km apart from each other (mean: 46.6 km). Plants were located in grassland fragments on slopes that were separated by fields, settlements and forests. Climate in this region is mild, with annual precipitation of 540 mm (interpolated climatic data based on [38]), cool summers (18°C mean temperature) and warm winters (−1°C). Soils in European populations consisted of nutrient poor sandy brown earths on glacial and periglacial sand deposits (see Table S1 for soil chemical properties). Asian populations, in the Naurzum district, Kostanayskaya Oblast, Kazakhstan (Figure 1C), were 9.6–69.6 km apart from each other (mean: 39.1 km) and covered flat plains with few settlements and forests in between. Climate in this region is dry with 260 mm annual precipitation, warm during the summer (22°C) and cold in the winter (−13°C). Soils were comprised of nutrient poor sandy chestnut soils on sand and loam sediments (see Table S1 for soil chemical properties).

### Collection of Soil and Seeds

Soil and seeds were collected in September 2008 within 10×10 m plots; each considered as representing a population. For each population, we collected ca. 100 g soil with a metal corer (diameter: 2 cm, length: 20 cm) from underneath six randomly chosen tussocks, respectively, thus obtaining a total of approximately 600 g of soil for each population. The soil corer was thoroughly washed with water before collecting soil from another population. We collected and pooled seeds from all individuals within each plot. Soil was air-dried and transported overland in closed plastic bags to the University of Halle-Wittenberg, Germany. The soil was sieved (2 mm mesh), roots were cut into 1 cm long pieces and this substrate was refrigerated at 5°C before further processing. Seeds were also stored at 5°C.

### General Experimental Setup

For our greenhouse experiment, we used a sterilized (autoclaved at 121°C three times for 1 h) 1∶4 sand∶loam mixture as background soil. By using a sterile background soil, we minimized the presence of contaminant soil biota in our pots and were able to provide equal soil abiotic growing conditions for our study plants. Seeds were surface sterilized (2 min in 50% sodium hypochlorite bleach, 2 min in 70% alcohol) to eliminate adherent soil biota. We pre-germinated seeds on heat sterilized (36 h, 200°C) sand and planted individual seedlings into 450 ml cone-tainer pots (Stuewe and Sons Inc., Corvallis, OR, USA). Similar cone-tainer pots were used in subsequent experiments. To avoid cross-contamination over the course of our experiment, we sterilized all equipment by flame or by soaking in bleach for one hour.

The experiment was conducted at the greenhouse facilities of the University of Halle-Wittenberg, from March to October 2009. Before conducting Experiments 1–3, we trained soil to build up local soil biota in the sterile background soil. By choosing this approach we avoided maternal effects of the soil biota and equalized abiotic differences among soil samples [39]. For this training stage, we planted ten seedlings from each of the ten populations, with one seedling per pot containing a 9∶1 mixture of sterilized background and field-collected soil from their own population. We watered plants once or twice a week individually using equal amounts of deionized water, being cautious to avoid leaching. After 13 weeks, we removed the above ground biomass and mixed the trained soil including chopped roots into one bulk sample for each respective population. Trained soil was later used as inoculum (15% per pot) aimed at testing general soil biota effects and local adaptation in plants. Using whole soil as inoculum is a common tool in soil biota studies (see [10], [13], [40] for similar approaches) given the technical challenge of teasing apart the effects of a large variety of different organisms in the soil [3], [41]. Moreover, this strategy allows studying overall soil biota effects on plants similar to what plants experience in the field [2], [13]. Using a low inoculum dosage excluded the confounding effects of abiotic soil properties, a tactic similarly used by Bever [40].

### Experiment 1: General Soil Biota Effect

The first treatment aimed to test the general effect of soil biota on plant growth in all populations. We grew plants in sterilized background soil either with soil inoculum from their own population (‘home soil’) or without soil inoculum (‘control soil’). A total of 200 pots were used: 2 treatments×5 populations×2 regions×10 replicates. All pots were distributed randomly on the greenhouse bench using 28 supports that were rotated every third week. To avoid contamination, pots were arranged in a chess-like manner within each support and watered individually to avoid splash transfer. Initially, two seedlings were planted. After three weeks, we removed the smaller seedling and started measuring the maximum height of the remaining plant at three week intervals to estimate growth rate. After 19 weeks, we harvested the shoot and root biomass. Aboveground material was dried in an oven at 80°C for 48 h. Roots were rinsed, dried in silica gel and stored in plastic bags with silica gel. For each plant, we measured dry weight (shoot and root). Furthermore, we quantified mycorrhizal colonization, including arbuscules, in roots of five plants per population that were grown in their ‘home soil’ or in ‘control soil’ according to the method by Trouvelot et al. [42].

### Experiment 2: Local Adaptation Within Regions

The purpose of the second treatment was to test for local adaptation within the two regions, respectively. We compared fully crossed plant - soil inoculum combinations from different source populations within the regions by growing plants in increasingly distant ‘away soils’. This approach allowed testing for local adaptation in a clinal manner by evaluating plant performance as a function of the continuous distance between plant and soil inoculum origin [25]. We used plants grown in ‘home soil’ from Experiment 1 for the distance of 0 km and grew an additional number of 400 plants: 4 treatments ( = 4 additional distances)×5 populations×2 regions×10 replicates. Planting of seedlings, handling of the experiment and harvest were as in Experiment 1.

### Experiment 3: Local Adaptation Between Regions

In a third treatment, we tested adaptation between Europe and Asia, by comparing growth of plants in ‘home soil’ and ‘away soil’ from the other region. In the latter case, we did not fully cross all plant and soil populations but for each plant population chose randomly (without replacement) soil from one population from the other region as soil inoculum. We used the following pairs: EU1/KZ3, EU2/KZ6, EU4/KZ7, EU5/KZ2, and EU6/KZ1. We used plants grown in ‘home soil’ from Experiment 1 and compared them to 100 additional ‘away soil’ pots: 5 populations×2 regions×10 replicates. Planting of seedlings, handling of the experiment and harvest were as in Experiment 1.

### Statistical Analysis

Soil biota effects can be analyzed in two ways, using either a net soil biota effect index or original dry weight values. Because both approaches have advantages and disadvantages [39], we used both methods in our analysis.

First, we performed an analysis with a net soil biota effect index similar to the index of relative interaction intensity in Armas et al. [43] as (X_NS_−X_S_)/(X_NS_+X_S_), with X_NS_ being the dry weight of a plant in non-sterile soil and X_S_ the dry weight of a plant in sterile soil. As we did not use paired sterile and non-sterile replicates, we employed a bootstrap procedure to estimate the net soil biota effect [44]. For that purpose, we calculated mean X_NS_ and mean X_S_ based on 10 bootstrapped samples, respectively, in the boot package [45] in R [46]. These values were incorporated to calculate a net soil biota effect. We repeated this calculation 999 times. We used a t-test to compare the general effect of soil biota between regions (Experiment 1). Local adaptation within regions was tested with linear mixed-effects models using the nlme package in R [47], with mean net soil biota effect as a response variable, distance and region as fixed predictor variables (Experiment 2). As plant and soil populations were crossed in a fully reciprocal manner we were able to analyze local adaptation in plants growing in soil from increasingly distant populations, and in plants of different origins growing in a soil population [48]. Thus we incorporated plant or soil population as random factors, respectively, and tested whether their interaction with distance improved the model fit [49]. To analyze local adaptation between regions (Experiment 3), we used a linear generalized least squares model, with mean net soil biota effect as a response variable, region and soil treatment as predictor variables. Using linear mixed-effects models, we tested whether plant population and the interaction between plant population and soil treatment as random effects improved the model fit.

Secondly, we analyzed original dry weight per plant (sum of shoot and root weight) with linear mixed-effects models. Throughout all analyses, we used soil treatment, region and supports (block) as fixed effects. If the variable block and its interactions were not found to significantly improve the model they were removed from the final model. We used soil treatment as a categorical factor when analyzing the general effects of soil (‘home soil’ vs. ‘control soil’, Experiment 1) and local adaptation between regions (‘home soil’ vs. ‘away soil’ from the other region, Experiment 3). In the case of local adaptation within regions (Experiment 2), plant and soil populations were fully reciprocal among populations, so that distance between plant and soil populations (‘away soil’) was used as a continuous predictor and local adaptation was tested from two perspectives (see above). Model selection followed the methodology proposed by Zuur et al. [49]. Frequency of mycorrhizal fungi in the root system was calculated in the program MycoCalc according to the method proposed by Trouvelot et al. [42].

We used repeated measures analysis to analyze the influence of soil treatment and region on plant height during the first nine weeks of the experiment. We chose this time frame because the linear plant height – time relationship became asymptotic after this date across all treatments. We used generalized least squares and linear mixed-effects models in the package nlme, as implemented in the program R, to construct our models. In the case of local adaptation within regions (Experiment 2), we treated distance between soil and plant origin again as a continuous variable and analyzed data from two perspectives (see above). To account for possible temporal pseudoreplication and variance heterogeneity through time, we tested models with different correlation and variance-covariance structures by inspecting the AIC [50]. The autoregressive heterogeneous variance-covariance structure had the best model fit accounting for temporal correlation among individual measurements and different variances at each time point. The covariable block was not found to significantly improve the model and was omitted from the analysis.

*P* values of random effects were calculated by a likelihood ratio test and by comparing the model with and without the random effect [49].

## Results

### General Soil Biota Effect (Experiment 1)

**Figure 2:**
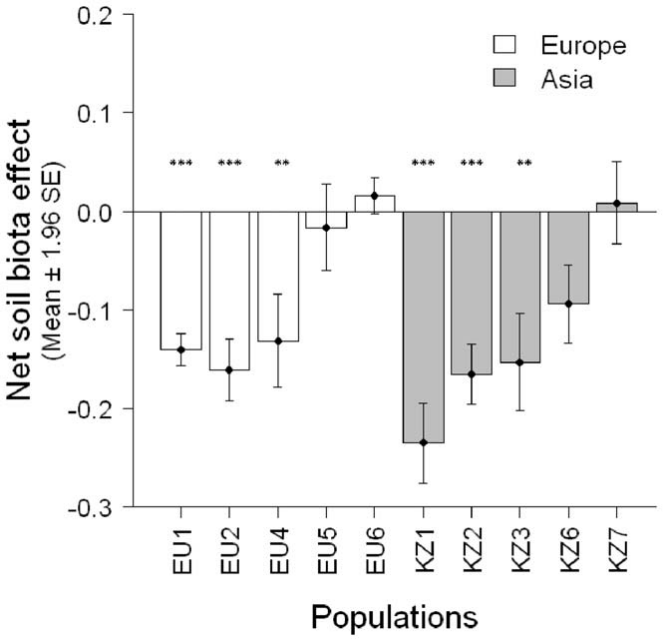
General soil biota effect in populations from Europe and Asia (Experiment 1). Bars represent estimated mean net soil biota effect, calculated as (X_NS_−X_S_)/(X_NS_+X_S_), with X_NS_ being the dry weight of a plant in home soil and X_S_ the dry weight of a plant in sterile control soil. As X_NS_ and X_S_ were not paired during the experiment, we used mean X_S_ and X_NS_ values based on 10 bootstrapped samples when calculating the net soil biota effect and repeated this procedure 999 times to estimate the mean net soil biota effect. Stars indicate whether in 95% (***) or 90% (**) of bootstrap runs effect values were different from zero. Mean net soil biota effect was not significantly different between Europe and Asia (t-test, *t* = 0.74, d.f. = 8, *P* = 0.480).

Net soil biota effect was negative in the majority of populations in Europe and Asia (Fig. 2). In each region, plants growing in ‘control soil’ produced significantly more dry weight than plants growing in ‘home soil’ (Fig. S1). Individuals from Europe produced generally more dry weight than those from Asia, but not for the ‘control soil’ (soil treatment * region, Fig. S1). Plants in ‘control soil’ also grew faster over the course of the first nine weeks compared to plants in ‘home soil’ (Time×soil treatment, Table S3). Roots of plants inoculated with ‘home soil’ had a significantly larger frequency of mycorrhizal colonization than those grown in ‘control soil’ (mean = 27.6% *vs.* 1.3%; Kruskal-Wallis Χ = 52.3, degrees of freedom (d.f.) = 1, *P*<0.001).

### Local Adaptation Within Regions (Experiment 2)

**Figure 3:**
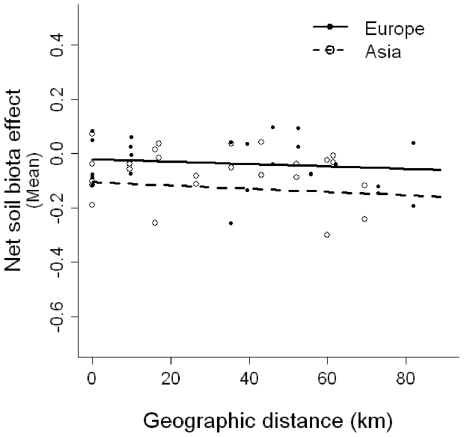
Estimated mean net soil biota effect as a function of geographic distance and region (Experiment 2). Points represent estimated mean net soil biota effect for Europe (solid) and Asia (open). Lines (solid for Europe and dashed for Asia) show the fitted linear mixed-effects model as predicted by the fixed effects geographic distance, region and their interaction for plants growing in soil from increasingly distant populations (random effects not shown). Geographic distance, region and their interaction were not found to significantly influence mean net soil biota effect (see Table 1A for details of the statistical test).

The net soil biota effect on plants did not change significantly with geographic distance, in both regions (Fig. 3, Table 1A). When comparing how plants from different populations performed in a given soil population, there was similarly no effect of geographic distance, region and their interaction (Table 1B). Dry weight (Fig. S2, Table S2) and growth rate (time×soil treatment, Table S4) did similarly not change significantly in response to geographic distance. Individuals from Europe produced generally more dry weight than those from Asia but this effect did not change with geographic distance (geographic distance×region, Table S2).

**Table 1:** Linear mixed-effects models to test for local adaptation within regions (Experiment 2).

	Effects	D.f.	*F*	*P*
A)	Plant population (random effect)			0.036
	Intercept	1, 38	38.61	**<0.001**
	Geographic distance	1, 38	1.20	0.282
	Region	1, 8	1.00	0.345
	Geographic distance×region	1, 38	0.06	0.810
B)	Soil population (random effect)			**0.007**
	Intercept	1, 38	31.39	**<0.001**
	Geographic distance	1, 38	1.42	0.241
	Region	1, 8	0.81	0.394
	Geographic distance×region	1, 38	0.22	0.645

Analyses were performed by inspecting the effects of the fixed variables geographic distance and region on estimated mean net soil biota effect. Geographic distance denotes the distance between the population of the plant and the soil inoculum. Models were constructed in two ways: A) from the perspective of plant populations growing in increasingly distant soil populations, by including plant population as a random effect, and B) from the perspective of soil populations in which plants from increasingly distant populations were grown, by including soil population as a random effect. *P* values≤0.01 are marked in bold.

### Local Adaptation Between Regions (Experiment 3)

**Figure 4:**
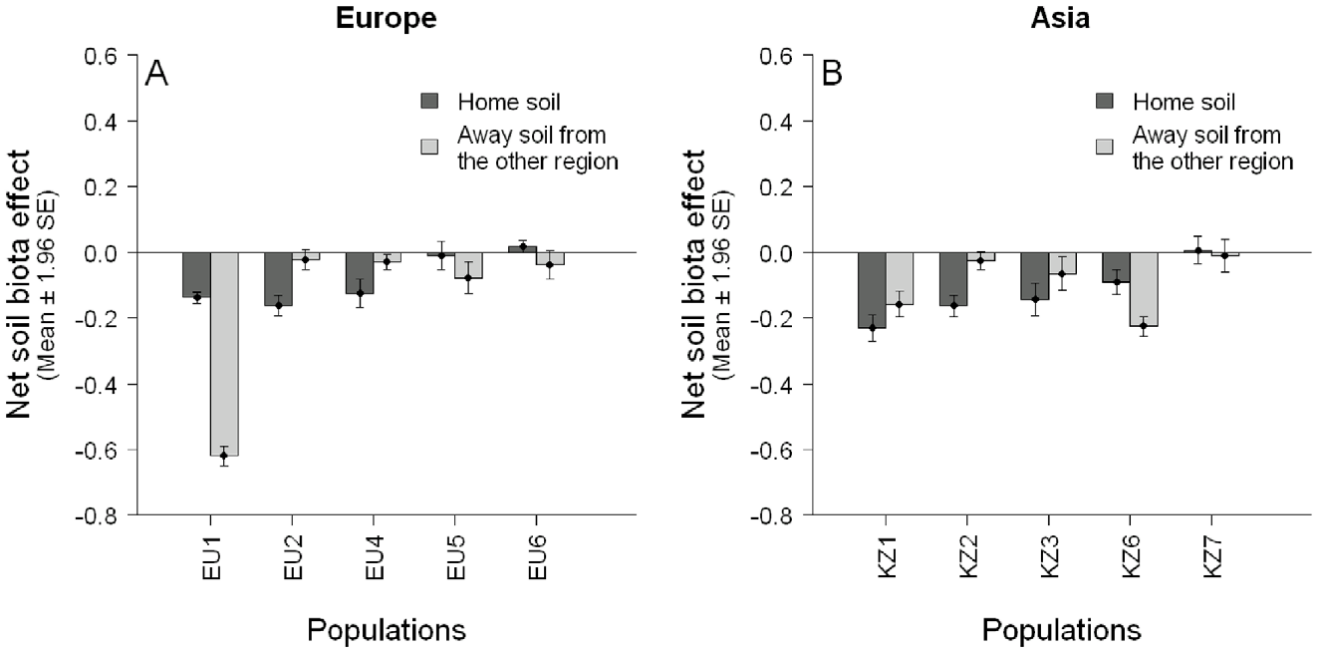
Mean net soil biota effect of plant populations in Europe and Asia grown in ‘home soil’ and ‘away soil’ from the other region (Experiment 3). Region, soil treatment and their interaction were not found to significantly influence mean net soil biota effect, as shown by a linear model: *F*_Region_ = 0.01, d.f. = 1,16, *P* = 0.912, *F*_Soil treatment_ = 0.11, d.f. = 1,16, *P* = 0.749, *F*_Region×soil treatment_ = 0.53, d.f. = 1,16, *P* = 0.475. Including plant population or the interaction of plant population with soil treatment as random effects in a linear mixed-effects model did not significantly improve the fit of the model.

There was no significant difference in estimated mean net soil biota effect when plants from Europe and Asia were grown in their ‘home soil’ or in ‘away soil’ from the other region (Fig. 4). Similarly, we did not find a significant difference in dry weight (Figure S3) and growth rate (Table S5) between plants grown in ‘home soil’ or ‘away soil’ from the other region.

## Discussion

### General Soil Biota Effect

Our study shows that *Stipa capillata* is controlled by negative soil biota effects: mean net soil biota effect was predominantly negative, dry weight and growth rate decreased significantly when plants were grown in their ‘home soil’ as compared to the sterile ‘control soil’. Thus, our study is consistent with a growing body of literature that reported negative soil biota effects on plants, especially grasses [2], [5], [8], [51]. Furthermore, our study is one of the first to show that negative soil biota effects can prevail in different parts of a species' range. A recent study by van Grunsven et al. [13] came to the same conclusion and showed that *Tragopogon pratensis* experienced negative soil biota effects throughout its range in Europe.

It is unlikely that our observed differences were caused by a higher proportion of background soil in control pots and thus by a higher nutrient content that arises from the process of sterilization. We minimized this putative confounding factor by keeping inoculum dosage low and pre-training our soil inoculum [40]. Thus, nutrients should have been similar across treatments. Furthermore, since inoculum dosage was low, it is unlikely that allelochemicals played a role in diminishing plant performance.

Our approach of using field collected whole soil as inoculum enabled testing for soil biota effects on plants as similar to field conditions as possible. It was not our goal to pinpoint the exact components of the soil biota responsible for the observed negative effect. However, our results call for future, more mechanistic, investigations to identify rhizosphere pathogens of grasses and their specific roles. Generally, many different groups of soil organisms can have a negative effect on plants belowground, including bacteria, protozoa, and nematodes [41]. Nevertheless, attempts to narrow down general soil organism groups responsible for negative effects have implicated fungi [5] and oomycetes in particular for being important [52].

### Local Adaptation Within and Between Regions

We found no evidence for local adaptation of *S. capillata* to soil biota. Plants grown in their ‘home soil’ and in ‘away soil’ did not experience a significantly different net soil biota effect index, dry weight, or growth rate. Contrary to our hypothesis, no local adaptation was detected at a larger scale, when plants were reciprocally transplanted between the two regions. This result is striking given that the study regions were 3000 km apart and differ drastically in macroclimatic properties that were likely to have favored different soil biota.

The lack of evidence for local adaptation in our experiment may be explained by several non-mutually exclusive causes. First, it is possible that soil biota composition was similar within and between the regions and, as a consequence, selection strength for locally adapted plants weak [53], [54]. Second, even if soil biota was differentiated across different localities [55], [56], such differences did not necessarily have to result in significant plant growth effects. Soil biota is compromised of a high diversity of species and strains [57], many of which might be functionally redundant [58]. Furthermore, past temporal variation in soil biota composition or even moderate migration rates could have enabled plants to respond to a variety of different soil organisms [32], [59]. Alternatively, it has been suggested that in species rich communities, coevolutionary responses are not governed by single pairwise interactions as much as by the diffuse interactions of multiple species [60], [61]. Thus, putative effects by some soil organisms on plant performance could have been overridden by other species [62]. From the plant perspective, gene flow among populations could have counteracted selection to local soil biota [32], [53], [54]. Although peripheral populations in Europe were genetically more differentiated than those in Asia (V. Wagner, W. Durka, I. Hensen, unpublished data) it is possible that even diminished gene flow was strong enough to counteract selection in this region. In addition, if genotype×genotype interactions are constrained by the environment (genotype×genotype×environment, [27], [32], [41], [63]), then experimental greenhouse conditions might have masked signs of local adaptation in our study. Therefore, future studies on local adaptation to soil biota should explore the role of environmental factors.

Plants can be locally adapted to a variety of environmental factors, including soil organisms [32]. Local adaptation is a significant consideration for land managers, for example when plant material is to be introduced to a target site. However, little has been done to test whether plant material needs to be derived from the same locality to perform best with soil biota at a restoration site. Our study provides a first indication that plants may not consistently be adapted to their local soil biota, so that this factor could potentially be precluded in management decisions. However, further studies are needed with other equally ecologically relevant plant species that take into consideration plant-plant competitive interactions [64], [65] as well as multiple abiotic factors.

### Conclusions

We found persistent negative soil biota effects in a widespread Eurasian grassland species, in two climatically contrasting parts of its native distribution range. Contrary to our hypothesis, we did not find evidence for local adaptation of *Stipa capillata* to soil biota, neither at the scale of 10–80 km, nor at the scale of 3300 km. Very little is known about coevolution and the roles of pathogen rhizosphere communities on their host plants and aboveground plant communities. Moreover, there is a need for further research that explores the role of environmental factors in soil biota effects, especially in a community context. Nevertheless, based on our data, when specifically considering possible effects of soil biota, managers should take into account that local adaptation to soil biota may not necessarily be present in plants.

## Supporting Information

Figure S1 **Dry weight of plants from populations in A) Europe and B) Asia grown in ‘home soil’ and in sterile ‘control soil’ (Experiment 1).** A linear mixed-effects model showed that soil treatment and region had a significant effect on dry weight: *F*_Soil treatment_ = 25.62, degrees of freedom (d.f.) = 1,187, *P*<0.001, *F*_Region_ = 5.63, d.f. = 1,8, *P* = 0.045, *F*_Soil treatment×region_ = 0.10, d.f. = 1,187, *P* = 0.748. Plant population was used as a random effect (*P*<0.001).
(TIF)

Figure S2 **Dry weight of plants as a function of geographic distance and region (Experiment 2).** Points represent dry weight of plants for Europe and Asia, respectively. Lines show the fitted linear mixed-effects model as predicted by the fixed effects geographic distance, region and their interaction for plants growing in soil from increasingly distant populations. Plant population was used as a random effect (lines not shown). Distance, region and their interaction were not found to significantly affect dry weight per plant (see Table S2 for details of the statistical test).
(TIF)

Figure S3 **Dry weight of plants from populations in A) Europe and B) Asia grown in ‘home soil’ and ‘away soil’ from the other region (Experiment 3).** A linear mixed-effects model showed that soil treatment, region and their interaction did not significantly influence dry weight production in plants: *F*_Soil treatment_ = 0.13, d.f. = 1,184, *P* = 0.718, *F*_Region_ = 4.8, d.f. = 1,8, *P* = 0.059, *F*_Soil treatment×region_ = 0.73, d.f. = 1,184, *P* = 0.393, *F*_Block_ = 4.0, d.f. = 1,184, *P* = 0.048. Plant population×soil treatment was used as a random effect (*P*<0.001).
(TIF)

Table S1 **Chemical properties of field collected soil and sterilized background soil used in the experiment.**
(DOC)

Table S2 **Linear mixed-effects models to test for local adaptation within regions (Experiment 2).** Dry weight was used as a function of the fixed effects geographic distance, region (and block). Geographic distance denotes the distance between the population of the plant and the soil inoculum. Models were constructed in two ways: A) from the perspective of plant populations growing in increasingly distant soil populations, by including plant population as a random effect, and B) from the perspective of soil populations in which plants from increasingly distant populations were grown, by including soil population as a random effect. *P* values≤0.01 are in bold.
(DOC)

Table S3 **Repeated measures analysis of plant growth during Experiment 1.** Plant height was measured on individuals every third week within a nine week period and used as a response variable of the fixed effects time, soil treatment (‘home soil’, ‘control soil’), region and their interaction. Including plant population or the interaction between plant population and time as random effects did not significantly improve the fit of the model. *P* values≤0.01 are in bold.
(DOC)

Table S4 **Repeated measures analysis of plant growth during Experiment 2.** Plant height was measured on individuals at the beginning and every third week of a nine week period and used as a response variable of the fixed effects time, geographic distance, and their interaction. Geographic distance denotes the distance between the population of the plant and the soil inoculum. We used population and individual nested within population as random factors. Models were constructed in two ways: A) from the perspective of plant populations growing in increasingly distant soil populations, by including plant population as a random effect, and B) from the perspective of soil populations in which plants from increasingly distant populations were grown, by including soil population as a random effect. *P* values≤0.01 are in bold.
(DOC)

Table S5 **Repeated measures analysis of plant growth during Experiment 3.** Plant height was measured on individuals at the beginning and every third week of a nine week period and used as a response variable of the fixed effects time, soil treatment (‘home soil’, ‘away soil’ from the other region), region and their interaction. Population and individual nested within population were used as a random intercept. *P* values≤0.01 are in bold.
(DOC)
