# Supplementary figures and images for: Prevailing Negative Soil Biota Effect and No Evidence for Local Adaptation in a Widespread Eurasian Grass
Source: PLoS One. 2011 Mar 29;6(3):e17580. doi: 10.1371/journal.pone.0017580 (PMC3066189; doi:10.1371/journal.pone.0017580)

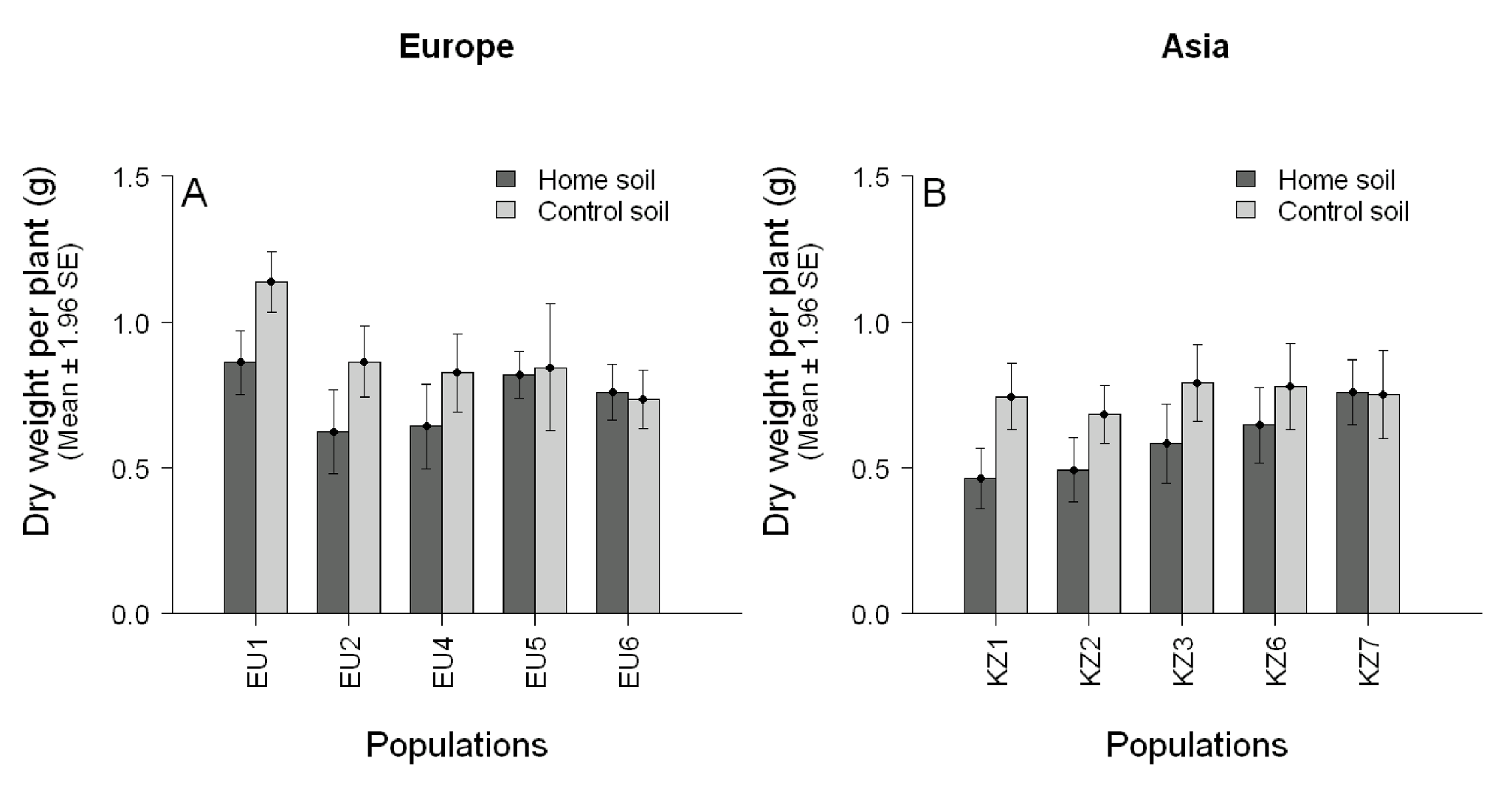

Supplement: Figure S1 — Dry weight of plants from populations in A) Europe and B) Asia grown in ‘home soil’ and in sterile ‘control soil’ (Experiment 1). A linear mixed-effects model showed that soil treatment and region had a significant effect on dry weight: F Soil treatment = 25.62, degrees of freedom (d.f.) = 1,187, P<0.001, F Region = 5.63, d.f. = 1,8, P = 0.045, F Soil treatment×region = 0.10, d.f. = 1,187, P = 0.748. Plant population was used as a random effect (P<0.001). (TIF) [file pone.0017580.s001.tif]

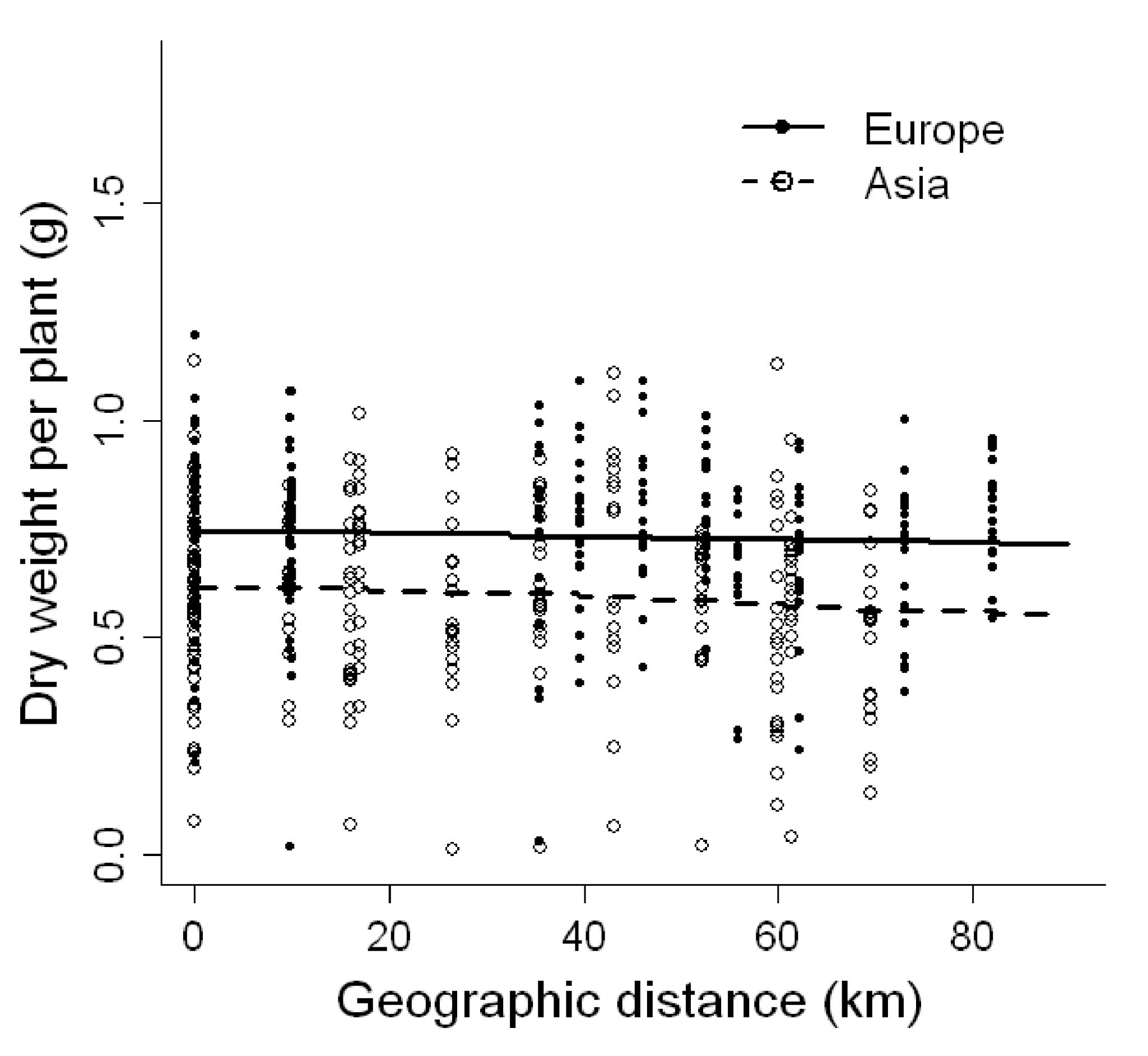

Supplement: Figure S2 — Dry weight of plants as a function of geographic distance and region (Experiment 2). Points represent dry weight of plants for Europe and Asia, respectively. Lines show the fitted linear mixed-effects model as predicted by the fixed effects geographic distance, region and their interaction for plants growing in soil from increasingly distant populations. Plant population was used as a random effect (lines not shown). Distance, region and their interaction were not found to significantly affect dry weight per plant (see Table S2 for details of the statistical test). (TIF) [file pone.0017580.s002.tif]

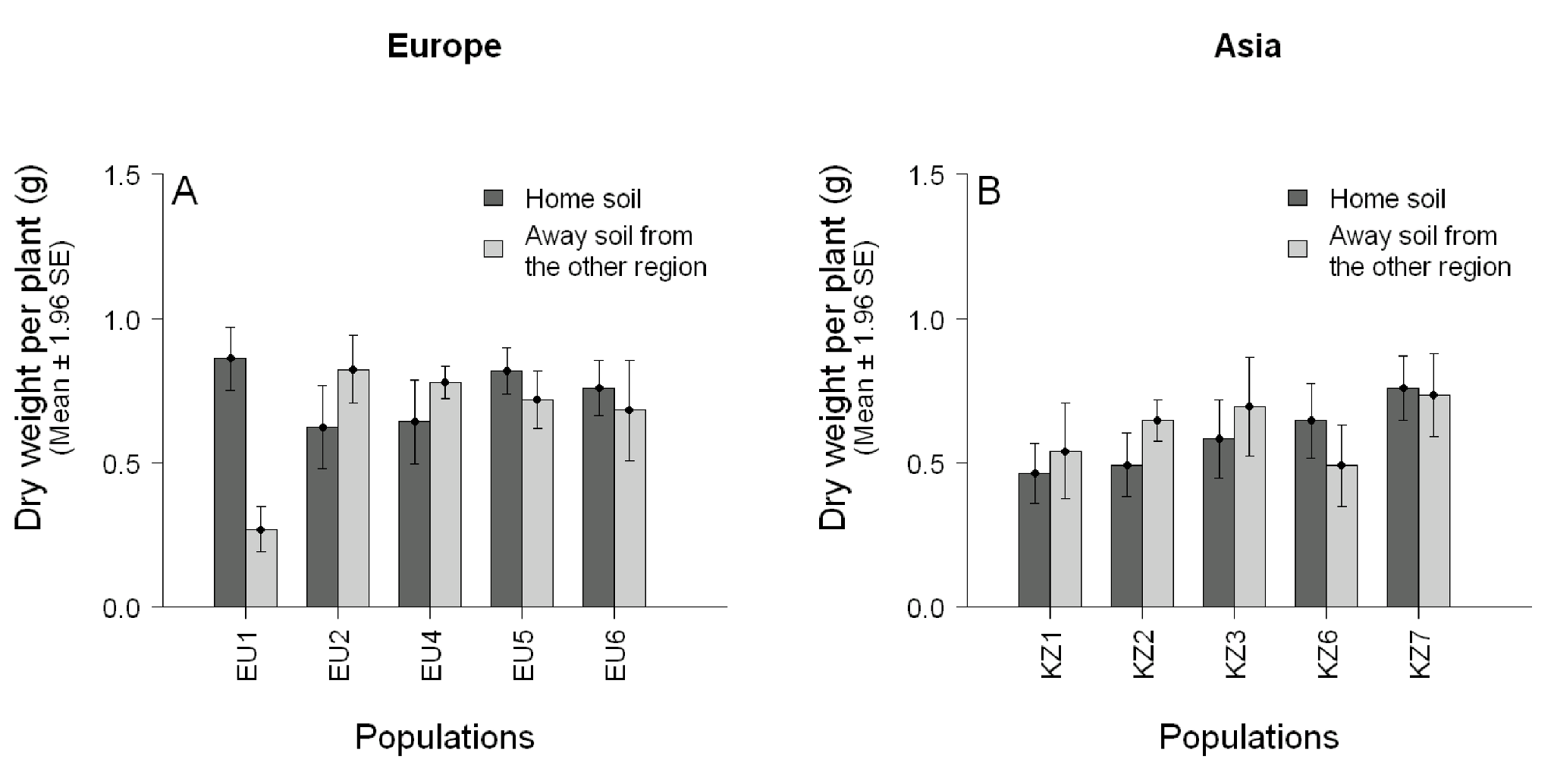

Supplement: Figure S3 — Dry weight of plants from populations in A) Europe and B) Asia grown in ‘home soil’ and ‘away soil’ from the other region (Experiment 3). A linear mixed-effects model showed that soil treatment, region and their interaction did not significantly influence dry weight production in plants: F Soil treatment = 0.13, d.f. = 1,184, P = 0.718, F Region = 4.8, d.f. = 1,8, P = 0.059, F Soil treatment×region = 0.73, d.f. = 1,184, P = 0.393, F Block = 4.0, d.f. = 1,184, P = 0.048. Plant population×soil treatment was used as a random effect (P<0.001). (TIF) [file pone.0017580.s003.tif]
