# Supplementary material for: Prevailing Negative Soil Biota Effect and No Evidence for Local Adaptation in a Widespread Eurasian Grass
Source: PLoS One. 2011 Mar 29;6(3):e17580. doi: 10.1371/journal.pone.0017580 (PMC3066189; doi:10.1371/journal.pone.0017580)
Supplement: Table S1 — Chemical properties of field collected soil and sterilized background soil used in the experiment. (DOC) [file pone.0017580.s004.doc]

| **Population** | **total N ^1)^ (µmol g^-1^)** | **total C ^1)^ (µmol g^-1^)** | **P ^2)^**  **(µg g^-1^)** | **pH ^3)^** | **Mg ^4)^ (mmol kg^-1^)** | **Ca ^4)^ (mmol kg^-1^)** | **K ^4)^ (mmol kg^-1^)** |
| --- | --- | --- | --- | --- | --- | --- | --- |
| **Asia:** | | | | | | | |
| **KZ 1** | 0.072 | 0.926 | 9.3 | 5.3 | 17.07 | 33.74 | 5.32 |
| **KZ 2** | 0.087 | 1.066 | 9.1 | 6.8 | 17.30 | 160.12 | 8.57 |
| **KZ 3** | 0.083 | 0.986 | 8.8 | 5.3 | 16.32 | 98.92 | 7.88 |
| **KZ 6** | 0.067 | 0.851 | 5.3 | 5.5 | 14.14 | 83.52 | 2.99 |
| **KZ 7** | 0.067 | 0.768 | 12.6 | 6.1 | 19.09 | 157.62 | 8.85 |
| **Mean:** | 0.075 | 0.919 | 9.0 | 5.8 | 16.78 | 106.78 | 6.72 |
| **Europe:** | | | | | | | |
| **EU 1** | 0.053 | 0.628 | 12.9 | 7.6 | 13.87 | 158.61 | 0.84 |
| **EU 2** | 0.067 | 0.875 | 13.6 | 7.7 | 14.32 | 188.32 | 1.98 |
| **EU 4** | 0.077 | 0.924 | 11.8 | 5.2 | 13.89 | 140.93 | 1.58 |
| **EU 5** | 0.135 | 2.133 | 8.9 | 7.6 | 15.17 | 298.45 | 2.82 |
| **EU 6** | 0.111 | 1.970 | 10.5 | 7.5 | 14.09 | 315.55 | 3.04 |
| **Mean:** | 0.090 | 1.310 | 11.5 | 7.1 | 14.27 | 220.37 | 2.05 |
| **Background soil:** | 0.037 | 0.646 | 70.6 | 7.6 | - | - | - |

^1)^ The content of total C and N was measured in finely ground soil using the combustion method in the Elemental Vario EL analyzer.

^2)^ The amount of available phosphorous was determined using the double lactate method by Egner and Riehm (1995).

^3)^ We measured soil pH in a solution of 10 g soil and 25 ml 1M KCl, after stirring the solution for 15 minutes.

^4)^ The amount of Mg, Ca and K was determined by percolation with BaCl_2_ and subsequent analysis in a flame atom absorption spectrometer (AAS vario 6, Analytik Jena).

Egner H, Riehm H (1955) Doppellaktatmethoden. In: Thun R, Herrmann R, Knickmann E, editors. Methodenbuch Bd I, Die Untersuchung von Böden. Radebeul and Berlin: Neumann Verlag.
