# Supplementary material for: Prevailing Negative Soil Biota Effect and No Evidence for Local Adaptation in a Widespread Eurasian Grass
Source: PLoS One. 2011 Mar 29;6(3):e17580. doi: 10.1371/journal.pone.0017580 (PMC3066189; doi:10.1371/journal.pone.0017580)
Supplement: Table S2 — Linear mixed-effects models to test for local adaptation within regions (Experiment 2). Dry weight was used as a function of the fixed effects geographic distance, region (and block). Geographic distance denotes the distance between the population of the plant and the soil inoculum. Models were constructed in two ways: A) from the perspective of plant populations growing in increasingly distant soil populations, by including plant population as a random effect, and B) from the perspective of soil populations in which plants from increasingly distant populations were grown, by including soil population as a random effect. P values≤0.01 are in bold. (DOC) [file pone.0017580.s005.doc]

|  | **Effect** | **D.f.** | ***F*** | ***P*** |
| --- | --- | --- | --- | --- |
| A) | Plant population x distance (random effect) |  |  | **<0.001** |
|  | Intercept | 1, 482 | 2595.07 | **<0.001** |
|  | Geographic distance | 1, 482 | 0.37 | 0.543 |
|  | Region | 1, 8 | 32.60 | **<0.001** |
|  | Geographic distance x region | 1, 482 | 0.19 | 0.660 |
| B) | Soil population (random effect)  (random effect) |  |  | **<0.001** |
|  | Intercept | 1, 481 | 755.98 | **< 0.001** |
|  | Geographic distance | 1, 481 | 0.52 | 0.471 |
|  | Region | 1, 8 | 8.08 | 0.022 |
|  | Block | 1, 481 | 7.00 | **0.008** |
|  | Geographic distance x region | 1, 481 | < 0.01 | 0.986 |
