# Supplementary material for: Prevailing Negative Soil Biota Effect and No Evidence for Local Adaptation in a Widespread Eurasian Grass
Source: PLoS One. 2011 Mar 29;6(3):e17580. doi: 10.1371/journal.pone.0017580 (PMC3066189; doi:10.1371/journal.pone.0017580)
Supplement: Table S3 — Repeated measures analysis of plant growth during Experiment 1. Plant height was measured on individuals every third week within a nine week period and used as a response variable of the fixed effects time, soil treatment (‘home soil’, ‘control soil’), region and their interaction. Including plant population or the interaction between plant population and time as random effects did not significantly improve the fit of the model. P values≤0.01 are in bold. (DOC) [file pone.0017580.s006.doc]

| **Effect** | **D.f.** | ***F*** | ***P*** |
| --- | --- | --- | --- |
| Individual x time (random effect) |  |  | **< 0.001** |
| Intercept | 1, 397 | 7012.33 | **< 0.001** |
| Time | 1, 397 | 3307.42 | **< 0.001** |
| Soil treatment | 1, 195 | 5.79 | 0.017 |
| Region | 1, 195 | 29.05 | **< 0.001** |
| Time x soil treatment | 1, 397 | 8.58 | **< 0.004** |
| Time x region | 1, 397 | 17.07 | **< 0.001** |
| Soil treatment x region | 1, 195 | 3.24 | 0.073 |
| Time x soil treatment x region | 1, 397 | 0.07 | 0.793 |
