# Supplementary material for: Prevailing Negative Soil Biota Effect and No Evidence for Local Adaptation in a Widespread Eurasian Grass
Source: PLoS One. 2011 Mar 29;6(3):e17580. doi: 10.1371/journal.pone.0017580 (PMC3066189; doi:10.1371/journal.pone.0017580)
Supplement: Table S4 — Repeated measures analysis of plant growth during Experiment 2. Plant height was measured on individuals at the beginning and every third week of a nine week period and used as a response variable of the fixed effects time, geographic distance, and their interaction. Geographic distance denotes the distance between the population of the plant and the soil inoculum. We used population and individual nested within population as random factors. Models were constructed in two ways: A) from the perspective of plant populations growing in increasingly distant soil populations, by including plant population as a random effect, and B) from the perspective of soil populations in which plants from increasingly distant populations were grown, by including soil population as a random effect. P values≤0.01 are in bold. (DOC) [file pone.0017580.s007.doc]

|  | **Effect** | **D.f.** | ***F*** | ***P*** |
| --- | --- | --- | --- | --- |
| A) | Individual x time (random effect) |  |  | **< 0.001** |
|  | Plant population x time (random effect) |  |  | **< 0.001** |
|  | Intercept | 1, 989 | 1226.00 | **< 0.001** |
|  | Time | 1, 989 | 1045.56 | **< 0.001** |
|  | Geographic distance | 1, 483 | 0.04 | 0.835 |
|  | Region | 1, 8 | 11.26 | **0.010** |
|  | Time x geographic distance | 1, 989 | 0.10 | 0.758 |
|  | Time x region | 1, 989 | 1.42 | 0.234 |
|  | Geographic distance x region | 1, 483 | < 0.01 | 0.994 |
|  | Time x geographic distance x region | 1, 989 | 0.11 | 0.743 |
| B) | Individual (random effect) |  |  | **<0.001** |
|  | Soil population (random effect) |  |  | **<0.001** |
|  | Individual (random effect) |  |  | **<0.001** |
|  | Intercept | 1, 989 | 4782.97 | **<0.001** |
|  | Time | 1, 989 | 6352.49 | **<0.001** |
|  | Geographic distance | 1, 483 | 0.14 | 0.713 |
|  | Region | 1, 8 | 32.118 | **0.001** |
|  | Time x geographic distance | 1, 989 | 0.22 | 0.641 |
|  | Time x region | 1, 989 | 10.14 | **0.002** |
|  | Geographic distance x region | 1, 483 | 1.59 | 0.208 |
|  | Time x geographic distance x region | 1, 989 | 0.04 | 0.847 |
