# Supplementary material for: Prevailing Negative Soil Biota Effect and No Evidence for Local Adaptation in a Widespread Eurasian Grass
Source: PLoS One. 2011 Mar 29;6(3):e17580. doi: 10.1371/journal.pone.0017580 (PMC3066189; doi:10.1371/journal.pone.0017580)
Supplement: Table S5 — Repeated measures analysis of plant growth during Experiment 3. Plant height was measured on individuals at the beginning and every third week of a nine week period and used as a response variable of the fixed effects time, soil treatment (‘home soil’, ‘away soil’ from the other region), region and their interaction. Population and individual nested within population were used as a random intercept. P values≤0.01 are in bold. (DOC) [file pone.0017580.s008.doc]

| **Effect** | **D.f.** | ***F*** | ***P*** |
| --- | --- | --- | --- |
| Plant population (random effect) |  |  | **< 0.001** |
| Individual (random effect) |  |  | **< 0.001** |
| Intercept | 1, 393 | 1012.37 | **< 0.001** |
| Time | 1, 393 | 2328.45 | **< 0.001** |
| Soil treatment | 1, 185 | 0.27 | 0.606 |
| Region | 1, 8 | 2.70 | 0.139 |
| Time x soil treatment | 1, 393 | < 0.01 | 0.968 |
| Time x region | 1, 393 | 8.64 | **0.004** |
| Soil treatment x region | 1, 185 | 9.06 | **0.003** |
| Time x soil treatment x region | 1, 393 | 1.19 | 0.275 |
